# Reduction of surface treatment time by combination of citric acid and ascorbic acid while restoring shear bond strength of metal brackets bonded to bleached enamel: a pilot study

**DOI:** 10.1186/s12903-024-04424-1

**Published:** 2024-06-12

**Authors:** Pichanee Saeoweiang, Pattraporn Chobpradit, Chadin Kulsing, Ekamon Mahapoka, Chanat Aonbangkhen, Thanit Charoenrat

**Affiliations:** 1 Department of Orthodontics, Chulalongkorn University, 34 Henri-Dunant Road, Bangkok, 10330 Thailand; 2 Department of Chemistry, Faculty of Science, Chulalongkorn University, Bangkok, 10330 Thailand; 3 Department of Operative Dentistry, Chulalongkorn University, Bangkok, 10330 Thailand; 4 Center of Excellence in Natural Products Chemistry (CENP), Department of Chemistry, Faculty of Science, Chulalongkorn University, Bangkok, 10330 Thailand; 5 Center of Excellence on Petrochemical and Materials Technology, Chulalongkorn University, Bangkok, 10330 Thailand

**Keywords:** Bleaching, Ascorbic acid, Citric acid, Antioxidant, Combined of citric acid and ascorbic acid, Shear bond strength and brackets

## Abstract

**Background:**

To investigate the effect of a 50% ascorbic acid with 50% citric acid solution on the immediate shear bond strength (SBS) of metallic brackets after tooth bleaching. The enamel etching pattern and the required quantity of these combined acids as antioxidants following 35% hydrogen peroxide (HP) bleaching were also determined.

**Methods:**

The stability of the solution at room temperature was assessed at various time intervals. Fifty teeth were randomly divided into five groups: non-bleached (G1), bleached then acid etched (G2), bleached followed by a 10-minute treatment with 10% sodium ascorbate and acid etched (G3), 5-minute treatment with 50% ascorbic acid (G4), and 5-minute treatment with a combination of 50% ascorbic acid and 50% citric acid (G5). Groups G2, G3, G4 and G5 were bleached by 35% HP gel for a total of 32 min. Acid etching in groups G1, G2, and G3 was performed using 37% phosphoric acid (Ormco®, Orange, CA, USA) for 15 s. In all groups, metal brackets were immediately bonded using Transbond™ XT primer and Transbond™ PLUS adhesive, with light curing for 40 s. The SBS was tested with a universal testing machine, and statistical analysis was conducted using one-way ANOVA followed by Tukey’s HSD test. The level of significance was set at *p* < 0.05 for all statistical tests.

**Results:**

Stability tests demonstrated that the combined acids remained effective for up to 21 days. Group G5 significantly increased the SBS of bleached teeth to the level of G1 (*p* < 0.05), while G3 did not achieve the same increase in SBS (*p* > 0.05). SEM analysis revealed enamel etching patterns similar to those of both control groups (G1 and G2). Kinetic studies at 6 min indicated that the antioxidation in G5 reacted 0.2 mmole lower than in G3 and G4.

**Conclusion:**

5-minute application of the combined acids enhanced the SBS of bleached teeth comparable to unbleached teeth. The combined acids remain stable over two weeks, presenting a time-efficient, single-step solution for antioxidant application and enamel etching in orthodontic bracket bonding.

**Supplementary Information:**

The online version contains supplementary material available at 10.1186/s12903-024-04424-1.

## Background

Due to the increasing demand for aesthetic dentistry, tooth whitening is a conservative and popular method for addressing the common concern of tooth discoloration [1, 2]. After tooth whitening treatments, patients can become more inclined towards having aesthetic procedures, such as veneers, composite restorations, or adjusting the alignment of mispositioned teeth through orthodontics. These interventions are all focused on enhancing the overall appearance and achieving a more satisfying aesthetic outcome for the patient [3–5]. Previous study shows that patients who received whitening treatment alongside orthodontic care reported increased satisfaction [6]. Patients who use aligners or ceramic brackets, as well as patients who are bonded with metal brackets, are seeking whitening before, during, or after treatment [7–10].

Different agents can be used for tooth bleaching. Hydrogen peroxide (HP) and carbamide peroxide are the most commonly used bleaching agents. However, tooth whitening, despite its advantages, can potentially reduce the shear bond strength (SBS) of orthodontic brackets. This effect is linked to the presence of oxygen free radicals that remain from the whitening process, including peroxides and superoxide anions. These peroxide molecules function by altering the chemical structure of organic compounds adhered to the enamel surface or present within the enamel. This process can consequently compromise tooth morphology and structure [11]. Furthermore, this process introduces the potential to interfere with the adhesive polymerization process, which is crucial for ensuring secure bracket bonding [12–15].

Various methods have been examined to enhance the bond strength after whitening procedures. It has been proposed to wait for a period ranging from 24 h to 3 weeks or more after whitening before performing the bonding process. This waiting period enables the gradual dissociation of peroxide free radicals from the tooth’s apatite structure, contributing to improved bond strength [16, 17].

As an alternative to postponing the bonding process, different approaches have been evaluated, including using adhesives containing organic solvents [18, 19], removing the superficial enamel [20], or applying antioxidants just before bonding [20–23]. The use of antioxidants after whitening has been identified as a way to restore the SBS of resin adhesive.

Numerous in vitro studies concerning applying antioxidants to enhance SBS have been performed [20, 22–25]. These studies included an additional etching procedure involving phosphoric acid, leading to an increase in the duration of the clinical procedure [20, 22, 24, 25]. A previous study proposed a method to improve the clinical effectiveness, reduce working time, and address this concern by combining antioxidant and acid etching solutions [23]. Notably, in vitro experiments have shown that tooth mineral loss can occur at pH 6.4 and below [21]. Hence, an antioxidant in an acidic form is favored to achieve this objective. In the present study, ascorbic acid was chosen due to its stability in aqueous solutions with a pH lower than 3.5 [22]. Furthermore, citric acid (CA) was selected as an alternative weak acid, because its prolonged working time [26] is suitable for enamel etching, coupled with its antioxidant properties.

The objective of this study was to explore the impact of combining ascorbic acid (AA) with CA on the immediate SBS of metallic brackets after bleaching. Additionally, the study assessed the enamel etching pattern and the required quantity of AA combined with CA, serving as an antioxidant agent following dental bleaching using 35% HP.

The null hypothesis was that surface treatment with AA combined with CA would have no effect on the SBS of the bleached human enamel surface and orthodontic metal brackets.

## Materials and methods

### Stock solution preparation

The stock solution of 50% AA in water was prepared by dissolving 0.5 g AA in 1 mL water, then heated at 40 ^o^C for 15 min. For the 50% CA stock solution, 0.5 g CA was dissolved in 1 mL water. The 10% sodium ascorbate (SA) in water was prepared by dissolving 0.1 g SA in 1 mL water. For the 50% ascorbic acid and 50% of citric acid (AACA) stock solution, 0.5 g AA and 0.5 g CA were mixed together in 1 mL water, which was heated at 40 ^o^C for 15 min to ensure complete solubility.

### Solvents and reagents

Potassium dihydrogen phosphate (KH_2_PO_4_) (CAS: 7778-77-0, Lot: K38125673 750), o-phosphoric acid (CAS: 7664-38-2, Lot: A850773 748) were sourced from Merck (Darmstadt, Germany), formic acid (CAS: 64-18-6, Lot: 1,358,336) was purchased from Fisher Scientific (Loughborough, UK).

### pH measurement

The pH of each sample stock solution was measured by a Mettler Toledo S220 pH meter.

### Stability test

**Fig. 1:**
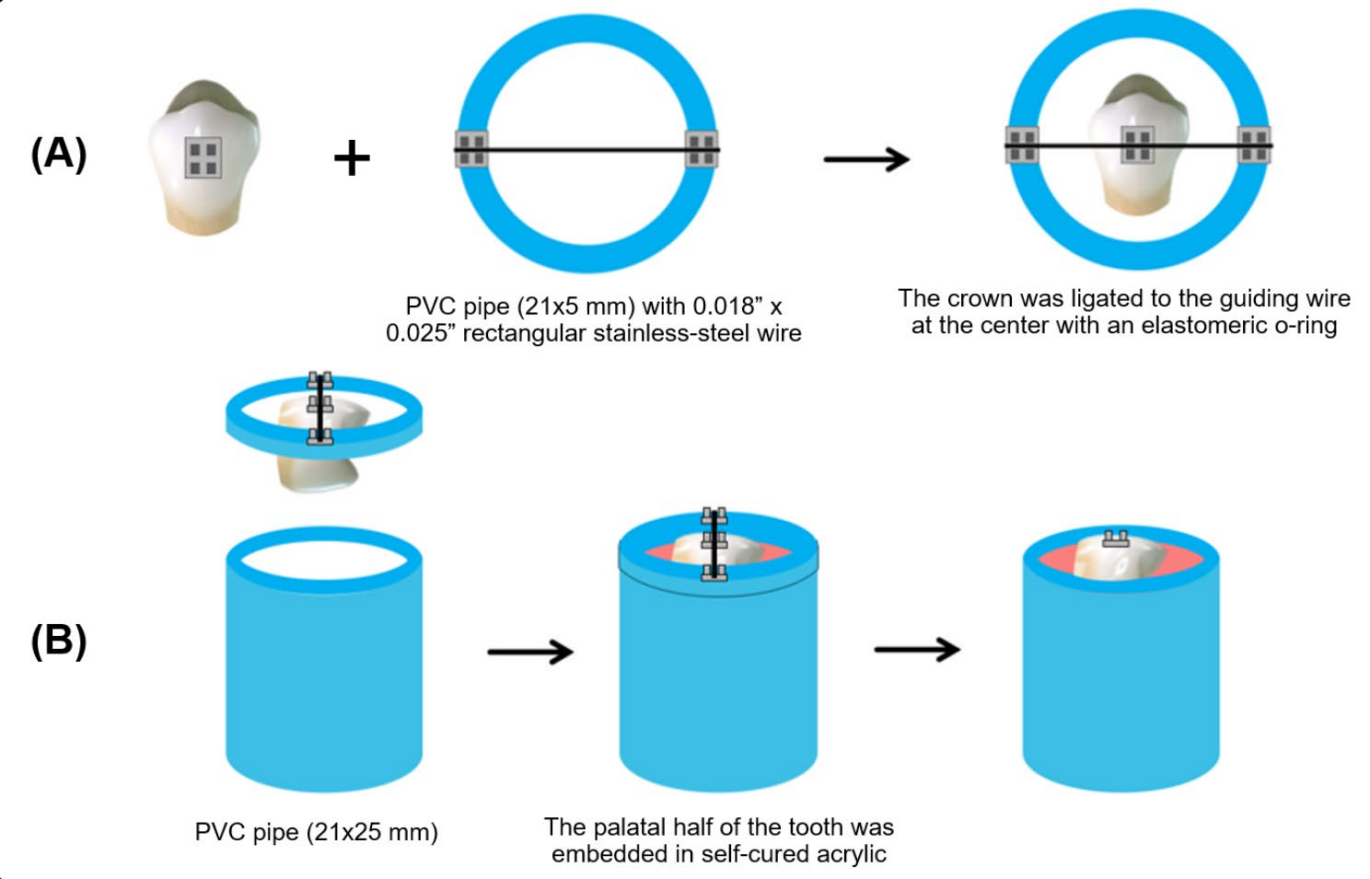
The illustration of Bonding index. **(A)** the prepared crown was ligated to the guiding index with an elastomeric o-ring at the center of the guiding wire. **(B)** the prepared crown attached to the guiding index was placed on a PVC pipe which contained self-cured acrylic. The palatal half of the tooth was embedded in self-cured acrylic at the established position

The stock solution of each sample was kept at room temperature (~ 30 ^o^C) for 0, 1, 3, 24, 48, 72 h and 7, 14, 21, 30 days. At each time point, 10 µL stock solution was dissolved with water to obtain 1 mL of the sample solution for HPLC analysis (Fig. 1(A)).

### Study design and sample preparation

G Power 3.1.9.7 software was used to calculate the sample size with the effect size of 1.014117 from a previous study [23] and power of 0.95, which indicated that 25 teeth were required. However, 50 teeth were ultimately used in the study. The inclusion criteria were upper premolars extracted from 17 − 30-year-old orthodontic patients. The selected teeth needed to have an intact buccal enamel surface and not have any fillings, decalcification, dental caries, cavities, cracks, hypoplastic enamel, fluorosis, or abnormalities in the enamel structure. Additionally, teeth that had undergone acid etching, application of bonding agents, use of vanish, bleaching, or any other procedures that could potentially compromise the integrity of the enamel were excluded. Prior to the experiment, the teeth were disinfected with 10% formalin solution for 2 weeks. Subsequently, the debris was removed using a sickle scaler, and the enamel was polished with fluoride-free pumice. The tooth was decoronated 2 mm below the cemento-enamel junction, and the crown was stored in fluoride-free artificial saliva at 37 °C for one week. The 50 teeth were randomly assigned to 5 different subgroups (Table 1). In the control group (*n* = 10), the buccal enamel surface was polished using fluoride-free pumice paste, rinsed, and air-dried. Subsequently, a 5 mm. wide and high area on the enamel surface was etched with 37% phosphoric acid (Ormco®, Orange, CA, USA) for 15 s. The etched area was rinsed for 30 s and dried using oil-free compressed air for 10 s. A thin layer of Transbond™ XT primer (3 M Unitek, St. Paul, MN, USA) was applied full to the etching area with the applicator. Transbond™ PLUS adhesive (3 M Unitek, St. Paul, MN, USA) was applied fully to the base of the 0.018” x 0.025” slot metal bracket (Omi arch® Roth type, TOMY, Fuchu-city, Tokyo, Japan) which was then immediately placed on the FA point of the tooth. The bracket was applied with a 100 g force using a Dontrix gauge (Orthopli, Philadelphia, PA, USA), with excess adhesive extruding from all four sides of the bracket to ensure that the adhesive fully filled the bracket base. Excess composite would be removed with a fine explorer and light-cured for 40 s at an intensity of 2000 mW/cm² using a Mini LED SATELEC® Ortho curing light (Acteon, Mount Laurel, NJ, USA). Prior to bonding in each group, the curing light was tested using a radiometer (Demetron®, SDS Kerr, Orange, CA, USA). During the bracket bonding process, a bonding index (Fig. 1) [23] was used to position the tooth in an acrylic block, ensuring parallel alignment between the universal machine’s blade and the bracket margin. Before conducting the bond strength tests, the specimens were kept in artificial saliva at 37 °C for 24 h.

**Table 1:** Tooth sample groups and their pre-bonding treatments

Group	No. of tooth specimens	Tooth bleaching procedure	Surface treatment	Etching procedure
Control	10	None	None	37% PA^1^
Bleach	10	35% HP^2^	None	37% PA
SA	10	35% HP	10SA^3^ 10 min	37% PA
AA	10	35% HP	50AA^4^ 5 min	None
AACA	10	35% HP	50AA/ 50CA^5^ 5 minutes	None

^1^PA: Phosphoric acid; ^2^HP: Hydrogen peroxide; ^3^10SA: 10%Sodium Ascorbate; ^4^50AA: 50% ascorbic acid; ^5^50AA/ 50CA: 50% ascorbic acid and 50% citric acid

In the second group, bleach group (*n* = 10), the buccal enamel surface of the teeth was polished with a fluoride-free pumice paste, rinsed, and air-dried. Subsequently, the teeth were bleached using a 35% HP gel (Pola Office®, SDI, Bayswater, VA, Australia) for a total of 32 min, divided into four 8-minute sessions. Immediately after the bleaching process, the teeth were etched and a metal bracket was bonded to the enamel surface, following the same protocol as the control group.

In the third group, SA group (*n* = 10), the teeth were bleached following the same procedure as in the Bleach group. After bleaching, the teeth were then treated with the 10% SA solution for 10 min. Subsequently, the teeth were etched, and a metal bracket was bonded to the enamel surface, following the same protocol as the control group.

In the fourth group, (AA group) (*n* = 10), the teeth underwent the same bleaching procedure as in the Bleach group. Immediately after bleaching, the teeth were treated with the 50% AA solution for 5 min. Subsequently, the metal bracket was primed and bonded to the enamel surface, following the same protocol as the control group.

Finally, in the AACA group (*n* = 10), the teeth were also bleached using the same procedure as in the Bleach group. Immediately after bleaching, the teeth were treated with the 50% AA and 50% CA solution for 5 min. Following this treatment, the metal bracket was primed and bonded to the enamel surface, without the use of 37% phosphoric acid etching, following the same protocol as the control group.

### The shear bond strength (SBS) test

The SBS was measured using an EZ-S universal testing machine (SHIMADZU, Nakagyo-ku, Kyoto, Japan) equipped with a 500 N load cell. The test was performed at a crosshead speed of 1 mm/min, applying a force in the occluso-gingival direction that was parallel to the interface between the tooth’s surface and the bracket. The machine measured and captured the shear force in newtons (Ns). To determine the SBS in megapascals (MPa), the shear force was divided by the area of the bracket base, which was calculated as 12.28 mm^2^ in a previous study [23].

### Enamel etching pattern by a scanning electron microscope (SEM)

In addition, one selected specimen from each group were examined using a SEM (JSM 5410 LV, JEOL, Tokyo, Japan) at 5,000X magnification operated at 20.00 kV and a working distance of 10 mm.

### Kinetics of the reaction between HP (bleaching gel) and samples (antioxidants)

**Fig. 2:**
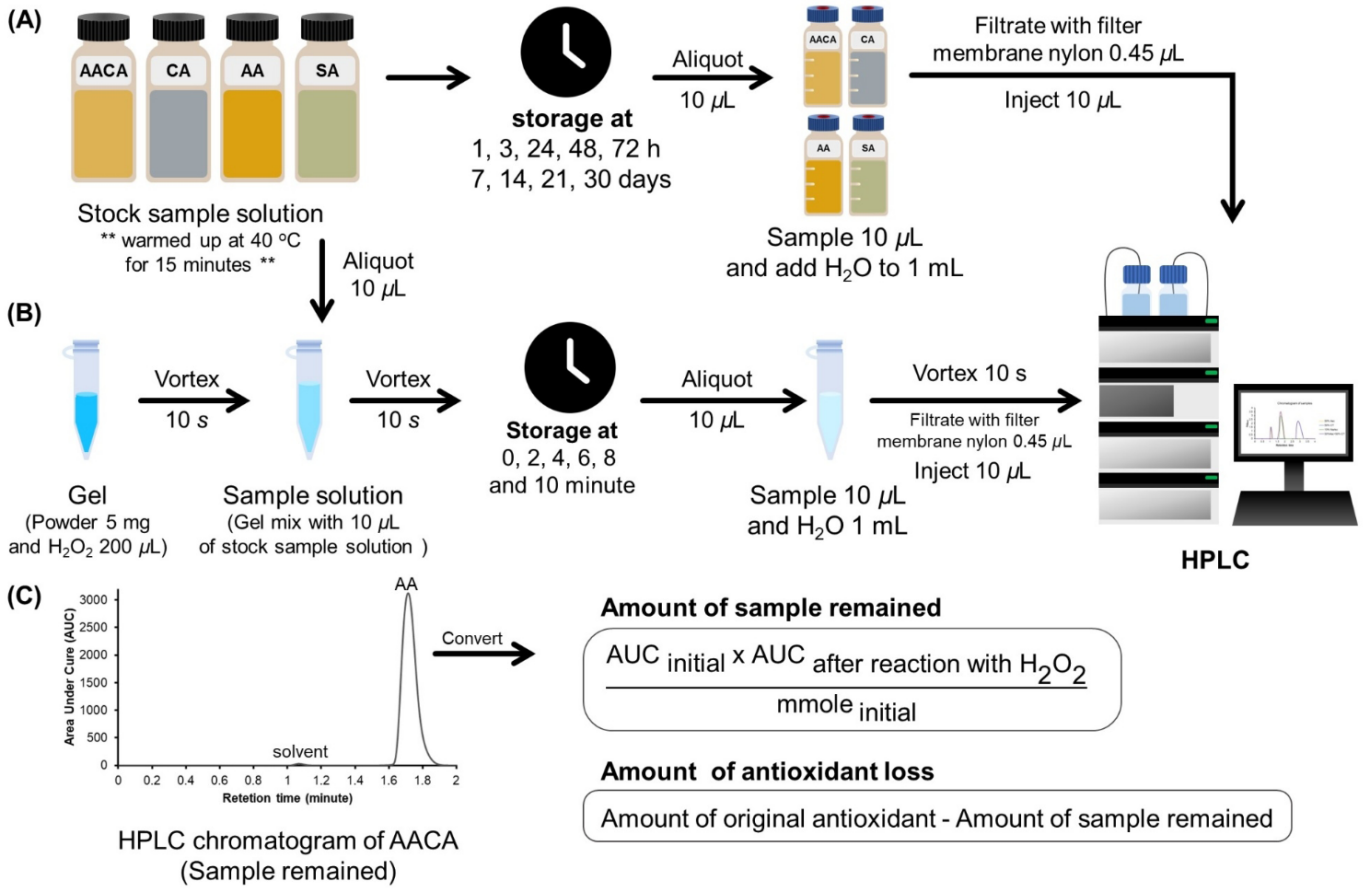
The experimental diagram of **(A)** stability test, **(B)** reaction kinetics and **(C)** HPLC chromatogram of AACA and calculation of antioxidant loss

The schematic diagram of the reaction kinetics of HP and antioxidants test is shown in Fig. 2(B). The bleaching gel was prepared by mixing 5 mg of the Pola office powder with an HP solution (35% v/v), which was then vortexed for 10 s to obtain the bleaching semi-gel solution. Next, 25 µL of each of the stock sample solutions, 50% AA, 50% AA and 50% CA or 10% SA, was mixed with 125 µL of the freshly prepared bleaching semi-gel solution, then vortexed for 10 s, and the reaction was allowed to proceed for 2, 4, 6, 8, 10 min and then every 5 min up to 1 h. After each time point, the solution was quickly vortex-mixed and a 10 µL aliquot was diluted in 1 mL water. Finally, 10 µL of the diluted sample was injected into the high-performance liquid chromatography (HPLC) instrument for the quantitative analysis of the reactants. Water was used instead of the 35% HP solution for measuring as a blank of each sample. The blank control contained only HP. The mass of each antioxidant’s loss was calculated from the amount of each antioxidant remaining in the solution after reacting with HP in the bleaching gel compared with the initial amount of antioxidant (Eq. 1 and Fig. 2C).

1 $$\eqalign{{\rm{Amount\,of\,antioxidant\,loss}}{\mkern 1mu} = {\mkern 1mu} & {\rm{Amount\,of\,original\,antioxidant}} \cr & - {\rm{Amount\,of\,sample\,remaining}} \cr}$$

### UV-HPLC analysis method

The stability test and the kinetics of the reaction between HP and the samples were performed on an Agilent HPLC 1260 Infinity II, the solution was separated by InfinityLab Poroshell 120 SB-C18 4.6 × 100 mm 2.7 μm column with a 1 mL min^− 1^ flow rate, the column temperature was set at 30 ^o^C and 10 µL of each sample was injected into the HPLC.

For the stability test, the mobile phase was prepared by dissolving 10 mM KH_2_PO_4_ in water, then o-phosphoric acid was used to adjust the pH to 2.2 [28], and the total time for analysis was set at 4 min. The UV detector was set at a wavelength of 210 nm. The area under the curve was measured, calculated using the blank or timepoint t = 0 min as the reference.

For the kinetics experiment, 0.1% formic acid in water was used as the mobile phase, the duration for chromatogram collection for this method was set at 2 min, and a wavelength of 254 nm was used for determining the remaining ascorbic acid/ ascorbate amount after reacting with the 35% HP solution in the bleaching gel at 2, 4, 6, 8 and 10 min.

### Statistical analysis

The data was processed using the SPSS software (version 22, statistical software). The normality of the data was assessed using the Kolmogorov-Smirnov test. To identify significant differences in SBS among the groups, one-way analysis of variance (ANOVA) was performed. Post hoc multiple comparisons were conducted using Tukey’s HSD test.

## Results

### Stability test

For the stability test, samples of the stock solutions, 50% AA, 50% CA, 10% SA, and 50% AACA, were stored at room temperature for 30 days to observe their stability at different durations. The amount of each sample was measured by HPLC at a wavelength of 254 nm. The peak area of each sample at various time points was converted to the percentage of the sample remaining compared with the initial content.

**Fig. 3:**
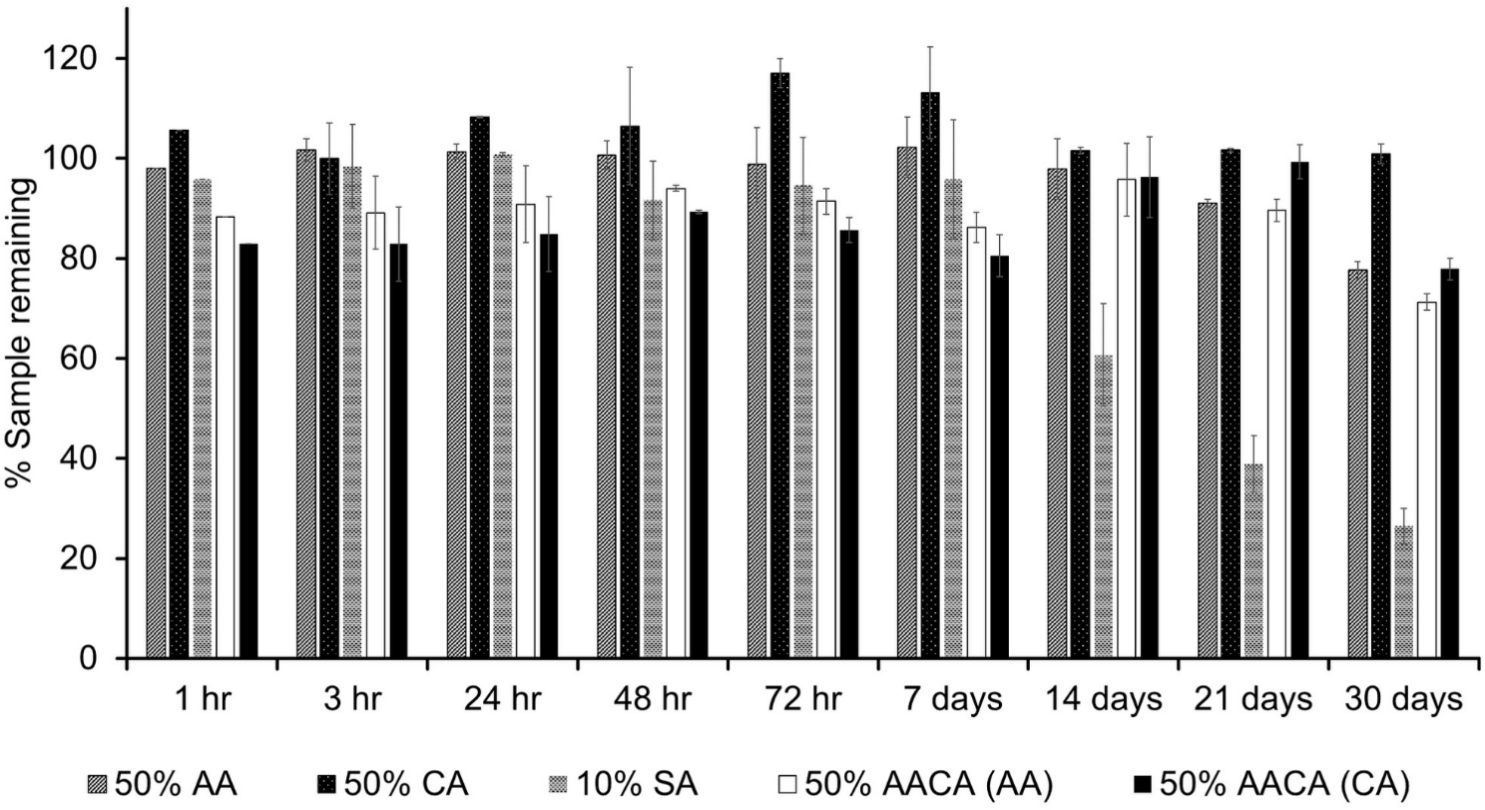
Stability test results; 50%AA: 50% ascorbic acid; 50%CA: 50% citric acid.; 10%SA: 10%Sodium Ascorbate; 50AA/ 50CA: 50% ascorbic acid and 50% citric acid

The stability test results are shown in Fig. 3. The content of each sample in the stock solutions was stable over 21 days, except for 10% SA, which demonstrated a significant decrease to 60% and 40% after 14 and 21 days of storage, respectively. The amount of 50% AA after storage at room temperature for 21 days remained over 90%, which then decreased to less than of the initial content after 30 days at room temperature. However, the amount of 50% CA was stable over 30 days and remained at approximately 99% of the original content.

The amount of 50% AA and 50% CA in the mixed solution were measured using the same method described above. The amount of AA and CA were stable for up to 21 days; the remaining content of each sample was more than 80% of the initial content in the mixed solution. After 30 days, the amount of AA and CA decreased to 71% and 77%, respectively, of the initial amount of each sample in the mixed solution.

### Enamel shear bond strength (SBS)

A significant difference in the mean SBS was observed among the test groups (*p* < 0.001 one-way ANOVA). The mean and standard deviation for each group is presented in Table 2.

The Tukey’s HSD test (Table 3) demonstrated that the SBS of brackets bonded immediately after bleaching enamel with 35% HP (bleach group) was significantly lower compared with that of brackets bonded with unbleached enamel (control group) (*p* < 0.001). However, when the 10% SA solution was applied, followed by 37% phosphoric acid etching (SA group), although the SBS increased, it was not significantly different from bleach group (*p* = 0.65). In contrast, there was a significant difference in SBS between the group that applied the 50% AA plus 50% CA formulation on bleached teeth for 5 min (AACA group) and bleach group (*p* < 0.05). However, AACA group showed no significant difference compared with SA group (*p* = 0.06) and the control group (*p* = 0.12). Furthermore, there was no significant increase in the SBS when 50% AA was applied on bleached teeth (AA group). SBS results for AA were similar to bleach group (*p* = 0.90) and not significantly lower than SA group (*p* = 0.065). However, the SBS in AA group was significantly lower compared with AACA group and control group (*p* < 0.05).

**Table 2:** pH of the samples

Sample	pH
37% Phosphoric acid	0.56
50% ascorbic acid/H_2_O	1.52
50% citric acid/H_2_O	0.90
10% sodium ascorbate/H_2_O	7.05
50% ascorbic acid + 50% citric acid/H_2_O	0.72
H_2_O	7.06

**Table 3:** The mean, standard deviation of the shear bond strength (MPa) for each treatment by group

Group*	Mean**	SD	Minimum	Maximum	Compare with	*p*-value
Control	20.94	4.90	10.35	29.18	Bleach SA AA AACA	< 0.001 < 0.001 < 0.001 0.119
Bleach	10.45	4.27	4.80	17.09	SA AA AACA	0.655 0.900 0.001
SA	12.65	4.50	4.38	19.00	AA AACA	0.692 0.065
AA	10.55	3.72	4.83	16.48	AACA	0.001
AACA	17.01	4.66	9.64	25.25		

*Control = 37% phosphoric acid on unbleached teeth for 15 s; bleach = 37% phosphoric acid on bleached teeth 15 s; SA = 10% sodium ascorbate treatment 10 min on bleached teeth and follow by 37% phosphoric acid 15 s; AA = 50% ascorbic acid on bleached teeth 5 min; AACA = 50% ascorbic acid plus 50% citric acid formulation on bleached teeth 5 min

**The One-way ANOVA test indicated that there were significant differences among the groups (*p* < 0.001)

### Enamel etching pattern by scanning electron microscopy (SEM)

**Fig. 4:**
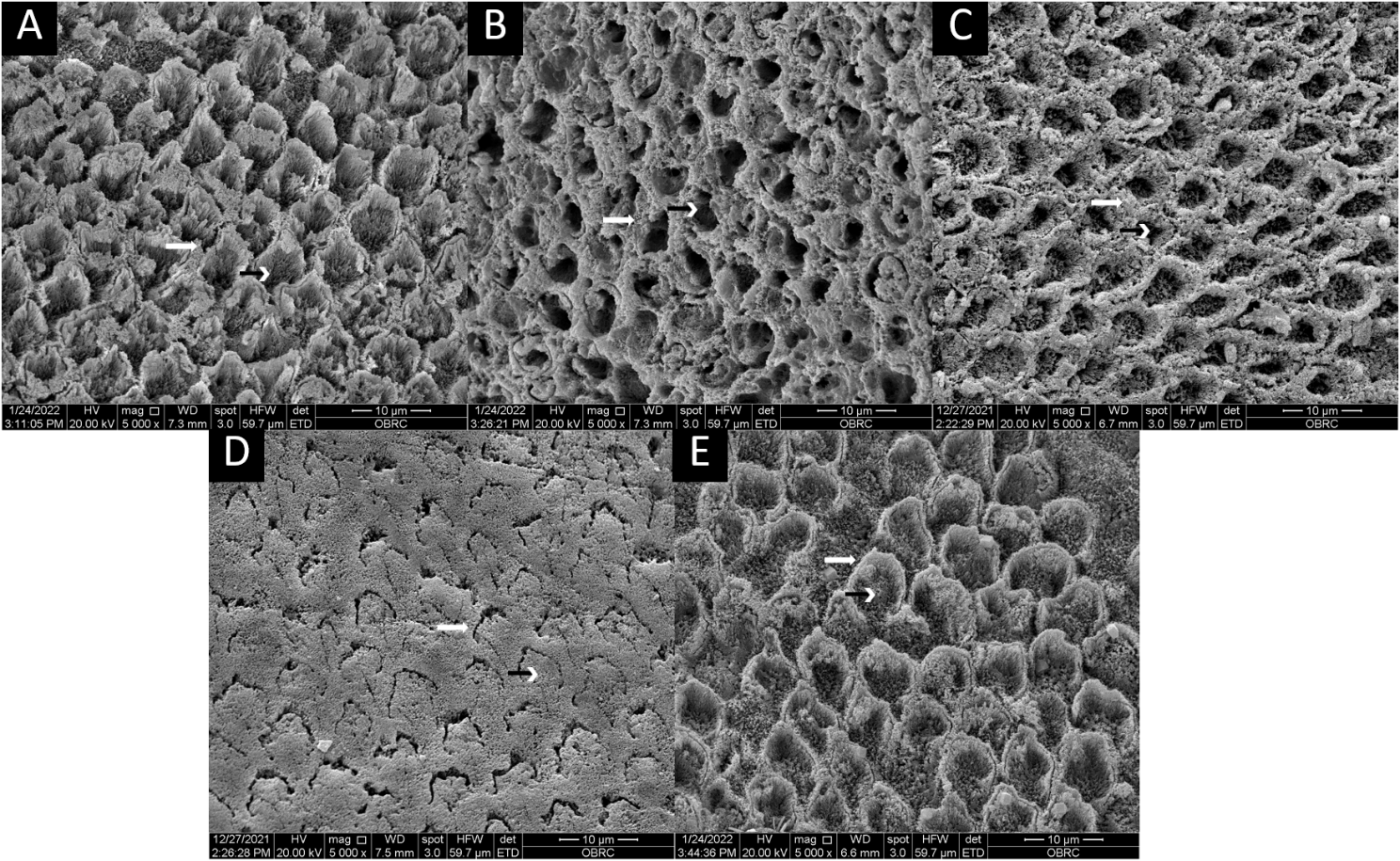
The scanning electron micrograph of the enamel surface after the bleaching agent and antioxidant treatment were applied. **A** = 37% phosphoric acid on unbleached teeth for 15 s; **B** = 37% phosphoric acid on bleached teeth 15 s; **C** = 10% sodium ascorbate treatment 10 min on bleached teeth and follow by 37% phosphoric acid 15 s; **D** = 50% ascorbic acid on bleached teeth 5 min; **E** = 50% ascorbic acid plus 50% citric acid formulation on bleached teeth 5 min

The scanning electron micrograph are displayed in Fig. 4. In the control group, the enamel prism core exhibited a prominent dissolution of the prism core while preserving the marginal area (Fig. 4A). A similar etching pattern was observed in bleach group (Fig. 4B), SA group (Fig. 4C), and AACA group (Fig. 4E). However, SA group, which used 35% HP before etching, exhibited the most irregular etching pattern. Interestingly, AA group, which used only 50% AA, resulted in the least profound etching pattern, predominantly affecting the prism periphery.

### Reaction kinetics between HP (bleaching gel) and samples (antioxidants)

**Fig. 5:**
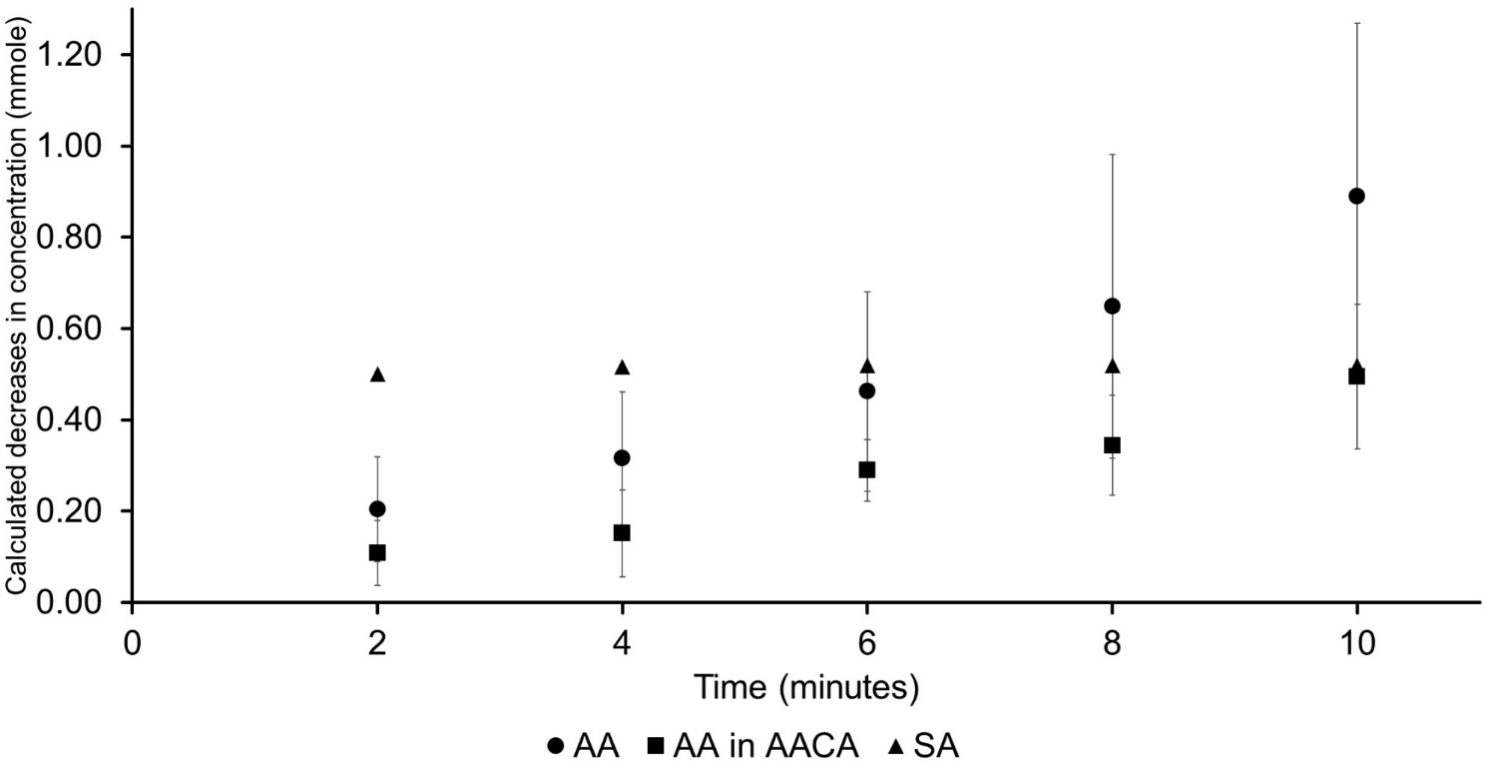
The reaction kinetics between hydrogen peroxide and 50% ascorbic acid, and 50% ascorbic acid in the mixed solution and 10% sodium ascorbate, with *p* < 0.05

The results of each antioxidant’s mass loss after reacting with bleaching gel (*p* < 0.05) is presented in Fig. 5.

The amount of each antioxidant continually decreased. The 10% SA showed no change in amount after reacting with the bleaching gel for 2 min. The amount of 50% AA in solution was oxidized by HP, with a mass loss of 0.20, 0.32, 0.46, 0.65 and 0.89 mmole every 2 min.

The 50% AA in the mixed solution presented a mass loss of 0.11, 0.15, 0.29, 0.34, 0.49 mmole every 2 min.

## Discussion

Based on our results, the first null hypothesis was rejected. The 50% CA and 50% AA combination reestablished SBS following the bleaching process to nearly that of the normal value. Although the surface treatment with 10% SA for 10 min followed with 37% phosphoric acid etchant increased SBS, it was not significantly different from the bleaching groups.

Prior studies [23, 27–29] found disparate effects of applying 10% SA to bleached teeth on restoring the SBS due to differences in methodologies, such as the bleaching gel concentration, bleaching time, SA application technique, and the form of SA. However, our study and other studies [23, 28, 29] found that 10% SA was insufficient to eliminate the high concentration of free radicals (high HP concentration) present after bleaching.

In line with other researches, using 35% HP for bleaching resulted in a significant reduction in SBS. This outcome is ascribed to the disruption caused by the free radicals in the infiltration process of the adhesive system, consequently impeding its polymerization [2, 30]. Furthermore, bleaching gels characterized by elevated HP concentration and reduced pH levels had negative effects on enamel morphology [30]. The findings of the present study revealed alterations in enamel morphology, with a greater loss of the prism cores compared with the prism peripheries, resulting in an irregular etching pattern in the bleached group. However, when teeth subjected to bleaching were treated with 10% SA, the etching pattern displayed a more consistent honeycomb-like appearance, resembling the typical pattern more closely than the group without any antioxidant treatment. Interestingly, when treated with a combination of 50% CA and 50% AA, the morphological alterations in enamel most closely resembled those observed in the non-bleached group. This observation indicated that the antioxidant concentration plays a crucial role in protecting the substrate from excessive mineral loss and aides in enamel remineralization [27, 31].

The selection of a 50% CA and 50% AA mixture for restoring the SBS of teeth bleached with HP was supported by the distinct advantages offered by each acid. AA, with its antioxidant properties, helped neutralize the free radicals created during bleaching. CA was recognized as an etchant for enamel and a stabilizer for AA [32]. The previous study [26] demonstrated that a 50% aqueous solution of CA, with a pH of 1.5 that used exceed 3 min, effectively etched enamel surfaces. This aligned with the antioxidant effect, which requires at least 5 min to react reductively with the HP in the bleaching agent [33]. However, the study chose not to use CA concentrations below 50%, considering the higher pH [34] which impacted the efficacy of the enamel etching process and could potentially extended operational times, contradicting the research objective to streamline procedures and reduce treatment duration.

Additionally, our investigation revealed that a 5-minute application of 50% AA alone was insufficient to restore the SBS. The SEM images revealed that 50% AA induces a shallower etching pattern than that produced by the other treatments, explained by the highest pH value (pH = 1.52) of 50% AA. In contrast, the lower pH of the 50% CA and 50% AA mixture (pH = 0.72) and phosphoric acid (pH = 0.2, per the manufacturer’s information) can induce alterations in the mineral content of highly mineralized substrates, causing the honeycomb-like appearance that creates the micromechanical lock between enamel and adhesive [35]. This was evidenced by the observed increase in SBS, which did not significantly differ from that of unbleached teeth, showing that 50% CA and 50% AA had different effects on SBS restoration.

The SEM images revealed distinct etching patterns in the enamel surfaces of the experimental groups. Among them, the control group and other treated groups showed differing levels of enamel prism core dissolution while maintaining the marginal area. Notably, the group treated with 35% HP before etching exhibited the most irregular etching pattern. The non-uniform etching pattern observed in the SEM images could lead to incomplete resin tag formation and hybridization [36]. However, some findings did not establish a clear connection between resin tag length and its impact on SBS [37–39]. A further study is recommended to gain more detailed insights into the depth of enamel loss and resin tag formation.

The reaction kinetics between the experimental antioxidants and HP were investigated. In the mixed solution. Only the mass of 50% AA was measured because CA does not react with HP due to its oxidation-reduction potential [40]. The results demonstrated that the 10% SA solution reached full reaction completion within 2 min. In contrast, the 50% AA solution, whether alone or in combination with CA, did not achieve complete reaction until 10 min had elapsed. However, at the 6-minute mark, the 50% AA solution exhibited a reaction rate similar to that of the 10% SA solution. Comparing the mixture of AA and CA mixture, it was noted that the reaction rate of AA was slightly lower, with a negligible difference of only 0.2 mmole. These findings suggest that the treatment time for the mixed solution can be reduced to 5 min to achieve the same results as the 10% SA application conducted over 10 min. However, the reaction kinetics conducted in a laboratory setting may not fully represent the complexities of the intraoral environment. One possible hypothesis for the reaction of the mixture solution containing CA suggests that it could potentially etch the enamel, leading to an increased surface area for AA to react with the ROS and the peroxide that remains in the bleached enamel. However, this phenomenon was not demonstrated in the reaction kinetics study. The higher SBS observed in the application of the mixture solution compared with the 10% SA at 10 min may be attributed to this effect.

The stability test results indicated that SA was less stable than the other acids. Additionally, the AA in the 50AA/ 50CA mixture demonstrated reduced stability. However, a previous study found that CA acts as a stabilizer to prevent oxidation, inducing AA degradation [32]. In the present study, AA degradation still occurred due to the high temperature [41] that the sample was prepared at to ensure complete solubility of the solution, however it still had > 80% stability over 21 days. The effect of duration, storage, and safety of the mixed solution on SBS should be investigated in future studies. Based on our results, the 50% CA and 50% AA mixture exhibited the potential to be used clinically to restore SBS of HP bleached teeth.

## Conclusion

Under the conditions of this in vitro study, applying the 50% AA/50% CA mixture for 5 min can significantly increase the immediate shear bond strength (SBS) of metallic bracket bonding to teeth bleached with 35% HP, which is not significantly different from the SBS of unbleached teeth or bleached enamel treated with 10% SA for 10 min. The etching pattern of the 50% AA/50% CA mixture showed a honeycomb pattern similar to 35% PA etching on unbleached teeth. In contrast, 50% AA alone cannot increase SBS in bleached teeth due to the lack of an acid etching effect. This formulation demonstrated better stability over 2 weeks than 10% SA. For 35% HP bleached teeth, the 50% AA/50% CA mixture applied for 5 min as a surface treatment and acid etch can replace the surface treatment using 10% SA for 10 min and 37% PA etching for SBS recovery.

### Electronic supplementary material

Below is the link to the electronic supplementary material.

Supplementary Material 1

## Data Availability

Data is provided in supplementary file.

## Abbreviations

SBS: Shear bond strength
HP: Hydrogen peroxide
SEM: Scanning electron microscope
PA: Phosphoric acid
10SA: 10%Sodium Ascorbate
50AA: 50% ascorbic acid
50AA/ 50CA: 50% ascorbic acid plus 50% citric acid
mg: Milligram
v/v: Volume per volume
HPLC: High-performance liquid chromatography
°C: Degree Celsius
KH_2_PO_4_: Potassium dihydrogen phosphate
mW/cm^2^: Milliwatts per square centimeter
MPa: Megapascals
kV: Kilovolt
mL min^-1^: Volume flow rate
min: Minute
mm: Millimeter
mm^2^: Square millimeter
mM: Millimolar
mmole: Millimole
µL: Microliter
µm: Micrometer
nm: Nanometer
sec: Second
