# Correction: Reduction of surface treatment time by combination of citric acid and ascorbic acid while restoring shear bond strength of metal brackets bonded to bleached enamel: a pilot study

**DOI:** 10.1186/s12903-024-04648-1

**Published:** 2024-08-01

**Authors:** Pichanee Saeoweiang, Pattraporn Chobpradit, Chadin Kulsing, Ekamon Mahapoka, Chanat Aonbangkhen, Thanit Charoenrat

**Affiliations:** 1 Department of Orthodontics, Chulalongkorn University, 34 Henri-Dunant Road, Bangkok, 10330 Thailand; 2 Department of Chemistry, Faculty of Science, Chulalongkorn University, Bangkok, 10330 Thailand; 3 Department of Operative Dentistry, Chulalongkorn University, Bangkok, 10330 Thailand; 4 Center of Excellence in Natural Products Chemistry (CENP), Department of Chemistry, Faculty of Science, Chulalongkorn University, Bangkok, 10330 Thailand; 5 Center of Excellence on Petrochemical and Materials Technology, Chulalongkorn University, Bangkok, 10330 Thailand

**Correction to: BMC Oral Health (2024) 24:680**

10.1186/s12903-024-04424-1

In this article [1], there was an error in the Acknowledgements section. The revised Acknowledgements is given below.
